# Simultaneously induced mutations in *eIF4E* genes by CRISPR/Cas9 enhance PVY resistance in tobacco

**DOI:** 10.1038/s41598-022-18923-0

**Published:** 2022-08-26

**Authors:** Ngoc Thu Le, Huyen Thi Tran, Thao Phuong Bui, Giang Thu Nguyen, Doai Van Nguyen, Dong Thi Ta, Duy Dinh Trinh, Attila Molnar, Ngoc Bich Pham, Ha Hoang Chu, Phat Tien Do

**Affiliations:** 1 Institute of Biotechnology, Vietnam Academy of Science and Technology, Hanoi, Vietnam; 2 Graduate University of Science and Technology, Vietnam Academy of Science and Technology, Hanoi, Vietnam; 3 Institute of Molecular Plant Sciences, University of Edinburgh, Edinburgh, UK

**Keywords:** Biotechnology, Plant sciences

## Abstract

Tobacco is an important commercial crop and a rich source of alkaloids for pharmaceutical and agricultural applications. However, its yield can be reduced by up to 70% due to virus infections, especially by a potyvirus *Potato virus Y* (PVY). The replication of PVY relies on host factors, and eukaryotic translation initiation factor 4Es (eIF4Es) have already been identified as recessive resistance genes against potyviruses in many plant species. To investigate the molecular basis of PVY resistance in the widely cultivated allotetraploid tobacco variety K326, we developed a dual guide RNA CRISPR/Cas9 system for combinatorial gene editing of two clades, eIF4E1 (*eIF4E1-S* and *eIF4E1-T*) and eIF4E2 (*eIF4E2-S* and *eIF4E2-T*) in the *eIF4E* gene family comprising six members in tobacco. We screened for CRISPR/Cas9-induced mutations by heteroduplex analysis and Sanger sequencing, and monitored PVY^O^ accumulation in virus challenged regenerated plants by DAS-ELISA both in T0 and T1 generations. We found that all T0 lines carrying targeted mutations in the *eIF4E1-S* gene displayed enhanced resistance to PVY^O^ confirming previous reports. More importantly, our combinatorial approach revealed that *eIF4E1-S* is necessary but not sufficient for complete PVY resistance. Only the quadruple mutants harboring loss-of-function mutations in *eIF4E1-S*, *eIF4E1-T*, *eIF4E2-S* and *eIF4E2-T* showed heritable high-level resistance to PVY^O^ in tobacco. Our work highlights the importance of understanding host factor redundancy in virus replication and provides a roadmap to generate virus resistance by combinatorial CRISPR/Cas9-mediated editing in non-model crop plants with complex genomes.

## Introduction

Tobacco (*Nicotiana tabacum*) is one of the widely cultivated nonfood cash crops, a natural pesticide and plant of pharmaceutical importance. It is also used as a model plant for studying gene functions and metabolic processes, and to produce high value chemicals, such as plant-based vaccines. However, the yield and quality of tobacco are heavily affected by virus infections, especially by *Potato virus Y* (PVY). PVY belongs to the *Potyvirus* genus in the *Potyviridae*, the largest family of plant-infecting RNA viruses^1^, and has a wide host range in the *Solanaceae* family including tomato, potato and tobacco^2–4^. According to the Cooperation Centre for Scientific Research Relative to Tobacco (CORESTA), PVY infection can result in up to 70% yield loss globally, which is associated with reduced plant performance (i.e., height, leaf size) and altered chemical composition of the infected leaves^5^. Thus, producing PVY resistant tobacco is of high importance.

The first PVY resistant tobacco line carrying mutations at the *va* locus on chromosome 21 was generated by X-ray mutagenesis^6^. Subsequently, three allelic forms (0, 1 and 2) of the recessive resistance gene *va*, conferring varying degrees of resistance to PVY, had been introduced into *Nicotiana tabacum* cultivars by traditional breeding methods^7^. Molecular characterization of the *va* locus revealed that *va* encodes for a eukaryotic translation initiation factor 4E (eIF4E)^8^, which is a component of the eIF4F pre-initiation complex. eIF4E can bind the 5′ m7G cap of mRNAs and other proteins including the 40S ribosomal subunit to promote the translation of endogenous gene transcripts^9^. In PVY-infected cells, eIF4E can also interact with a virus-derived and viral genome-linked protein called VPg to initiate the translation of viral RNAs^9,10^. Thus, eIF4E acts as a host factor for PVY infection. Recently, both *eIF4E* and its isoform *eIF(iso)4E* have been identified as recessive resistance genes against different potyvirus isolates in diverse plant species^9–15^. The lack of functional interactions between the viral VPg and the plant eIF4E/eIF(iso)4Es is the primary cause of potyvirus resistance in most cases. Conversely, the compatibility of these interactions likely determines the PVY virus host range^16^.

The expression of host factors can be suppressed by RNA interference (RNAi), and a recent work demonstrated that RNAi-mediated down regulation of *eIF4E* and *eIF(iso)4E* genes can result in a reduced susceptibility to PVY and resistance breaking (RB)-PVY strains in tobacco^17^. However, the efficacy of RNAi-based virus resistance is heavily influenced by several internal and external factors such as the level of transgene-derived short interfering RNAs (siRNA)^18,19^, the degree of homology between the RNAi trigger sequence and the target virus^20,21^, and the cell types^22^. In addition, the transgenic RNAi plants meet heavy regulatory challenges and limited public acceptance in many countries^23^. Field studies revealed that *va0*, *va1* and *va2-*mediated potyvirus resistance can also be overcome by different potyvirus strains^24–26^. Thus, a novel approach is required to generate a broad and long-lasting resistance against the most prevalent potyviruses.

The latest gene editing technology referred to as CRISPR/Cas has been extensively employed to improve agronomic traits including virus resistance^27,28^. CRISPR/Cas can be expressed as a transgene and subsequently programmed to recognize and cleave invading virus genomes having complementary sequences to Cas guide RNAs^29,30^. Alternatively, CRISPR/Cas can be used to generate loss of function mutations in viral host factors, such as eIF4E or eIF(iso)4E^31–38^. The latter strategy has already been successfully employed to create potyvirus resistance in different plant species including cucumber^12^, Arabidopsis^13^, tomato^35^, cassava^39^ and barley^40^.

In this study, we used a dual sgRNA-expressing CRISPR/Cas9 construct to knock out multiple copies of the *eIF4E* gene family in the tobacco cultivar K326. We found that the simultaneous deletion of four *eIF4E* genes including *eIF4E1-S*, *eIF4E1-T*, *eIF4E2-S* and *eIF4E2-T* resulted in durable resistance to PVY both in T0 and T1 generations.

## Results

### Analysis of the *eIF4E* gene family in tobacco variety K326 for CRISPR/Cas9 target selection

**Figure 1:**
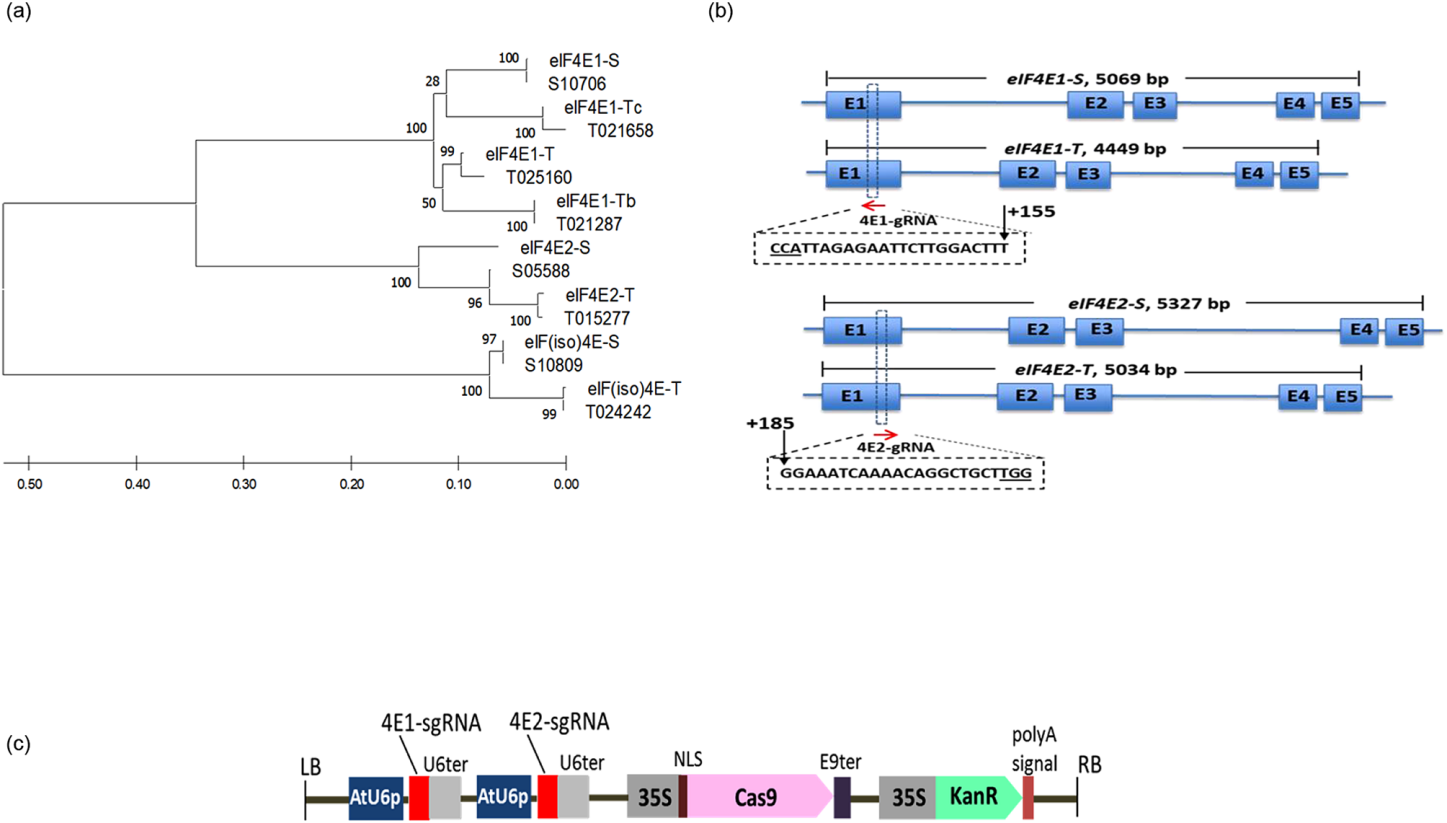
Tobacco eIF4E diversity, sgRNAs selection and CRISPR/Cas9 vector map. (**a**) Phylogenetic analysis of the eIF4E family in tobacco cultivar K326. The phylogenetic tree was constructed by the maximum likelihood method using the nucleotide alignment of genes encoding eukaryotic initiation factors in tobacco variety K326 along with reference sequences including the *N. tomentosiformis* (T) genome [T015277 (Genbank KM202067), T021658 (Genbank KM202068), T021287 (Genbank KM202069), T025160 (Genbank KM202070) and T024242 (Genbank KM202065)]; the *N. sylvestris* (S) genome [S10760 (Genbank KF155696), S05588 (Genbank KM202071), and S10809 (Genbank KM202066)]; and the K326 *eIF4E/eIF(iso)4E* genes [*eIF4E1-S*; *eIF4E1-T*; *eIF4E1-Tc*; *eIF4E1-Tb*; *eIF4E2-T*; *eIF4E2-S*, *eIF(iso)4E-S*; *eIF(iso)4E-T*]. Bootstrap values are shown above the corresponding branches. (**b**) Schematic of the *eIF4E* gene structures and the CRISPR/Cas9 target sites. The position and sequence of sgRNA target sites are indicated by a red arrow and a dashed rectangle, respectively. The sequences of the protospacer adjacent motifs (PAM) are underlined. The position of the sgRNA targeted region relative to the translation start site of the corresponding ORF is indicated by a black arrow. E1-E5, exons of *eIF4E* genes. (**c**) Schematic of the CRISPR/Cas9-sgRNA construct used for tobacco transformation. *KanR* Kanamycin resistant gene, *Cas9* Maize-codon-optimized Cas9 gene driven by the 35S promoter (Cauliflower Mosaic Virus promoter); two sgRNAs (4E1-sgRNA and 4E2-sgRNA) regulated by the Arabidopsis U6 promoter (AtU6p), *U6ter* Arabidopsis U6 terminator, *E9ter* Pea RuBisCO small subunit E9 terminator, *NLS* Nuclear localization signal, *LB/RB* left and right border.

Tobacco is an allotetraploid plant, which inherited its genome from the diploid *Nicotiana sylvestris* (S-genome) and *Nicotiana tomentosiformis* (T-genome)^41^. Detailed characterization of *eIF4E* and its isoform *eIF(iso)4E* in tobacco revealed that five of these genes are derived from the *N. tomentosiformis* (T) genome [T015277 (Genbank KM202067), T021658 (Genbank KM202068), T021287 (Genbank KM202069), T025160 (Genbank KM202070) and T024242 (Genbank KM202065)], and three are originated from the *N. sylvestris* (S) genome [S10760 (Genbank KF155696), S05588 (Genbank KM202071), and S10809 (Genbank KM202066)]^8^. To identify the eIF4E homologues in the *N. tabacum* K326 genome (https://solgenomics.net/organism/Nicotiana_tabacum/genome), we performed a TBlastN search using the amino acid sequence of a tobacco IF4E domain (GenBank accession number QNT12790.1) as a protein query. This exercise returned eight candidate sequences, all of which contained a typical IF4E domain as confirmed by Pfam analysis (http://pfam.xfam.org/). Phylogenic analysis revealed that the *eIF4E* genes form three well-separated clusters/clades in K326 (Fig. 1a). The first cluster corresponds to the eIF4E1 group and includes four genes: one from *N. sylvestris* designated as *eIF4E1-S* and three from *N. tomentosiformis* named as *eIF4E1-T*, *eIF4E1-Tb* and *eIF4E1-Tc*. The second eIF4E2 cluster contains one orthologous gene from each ancestor: *eIF4E2-S* and *eIF4E2-T*. The third cluster includes two eIF(iso)4E sequences, *eIF(iso)4E-S* and *eIF(iso)4E-T*.

Redundancy in host/susceptibility factor genes, such as *eIF4E*, is a major obstacle in generating virus resistance because the invading viruses could recruit a plethora of paralog gene products to support their life cycle^42^. Consequently, mutations in only one member of the *eIF4E* gene family may result in a non-durable, and limited virus resistance spectrum to PVY^17,43^. In contrast, deletion of all *eIF4E* genes could be associated with severe developmental defects or lethality due to the fundamental role of eIF4Es in the translation of endogenous mRNAs^44,45^. We speculated that knocking out multiple, but not all *eIF4E* genes could result in a durable and broad resistance against PVY without jeopardizing plant performance. In the eIF4E1 clade (Fig. 1a), *eIF4E1-Tb* has not been implicated in PVY resistance yet. However, the expression level of *eIF4E1-Tc* has been shown to positively correlate with durable PVY resistance in tobacco cultivars^43^. RNAi-mediated suppression or targeted mutagenesis of the remaining eIF4E1 members *eIF4E1-S* and *eIF4E1-T*, and the eIF4E2 clade members *eIF4E2-S* and *eIF4E2-T* were associated with PVY resistance^17,43,46^ but the additive and combined effects of the above genes on PVY susceptibility have not been investigated. Hence, we decided to simultaneously knock out *eIF4E1-S*, *eIF4E1-T*, *eIF4E2-S* and *eIF4E2-T* by CRISPR/Cas9, and subsequently investigate the effect of the combinatorial mutations on PVY susceptibility in tobacco K326. To this end, we designed two Cas9 guide RNAs referred to as 4E1-sgRNA and 4E2-sgRNA, which can independently target the *eIF4E1* (*eIF4E1-S* and *eIF4E1-T*) and *eIF4E2* (*eIF4E2-S* and *eIF4E2-T*) orthologues, respectively (Fig. 1b, Supplementary Fig. S1), but not the *eIF4E1-Tb* and *eIF4E1-Tc* (Supplementary Fig. S2). We then generated a dual CRISPR/Cas9 construct harboring both the 4E1-sgRNA and 4E2-sgRNA expression cassettes for tobacco transformation (Fig. 1c).

### Generation of transgenic tobacco lines and the analysis of CRISPR/Cas9-induced mutations

**Figure 2:**
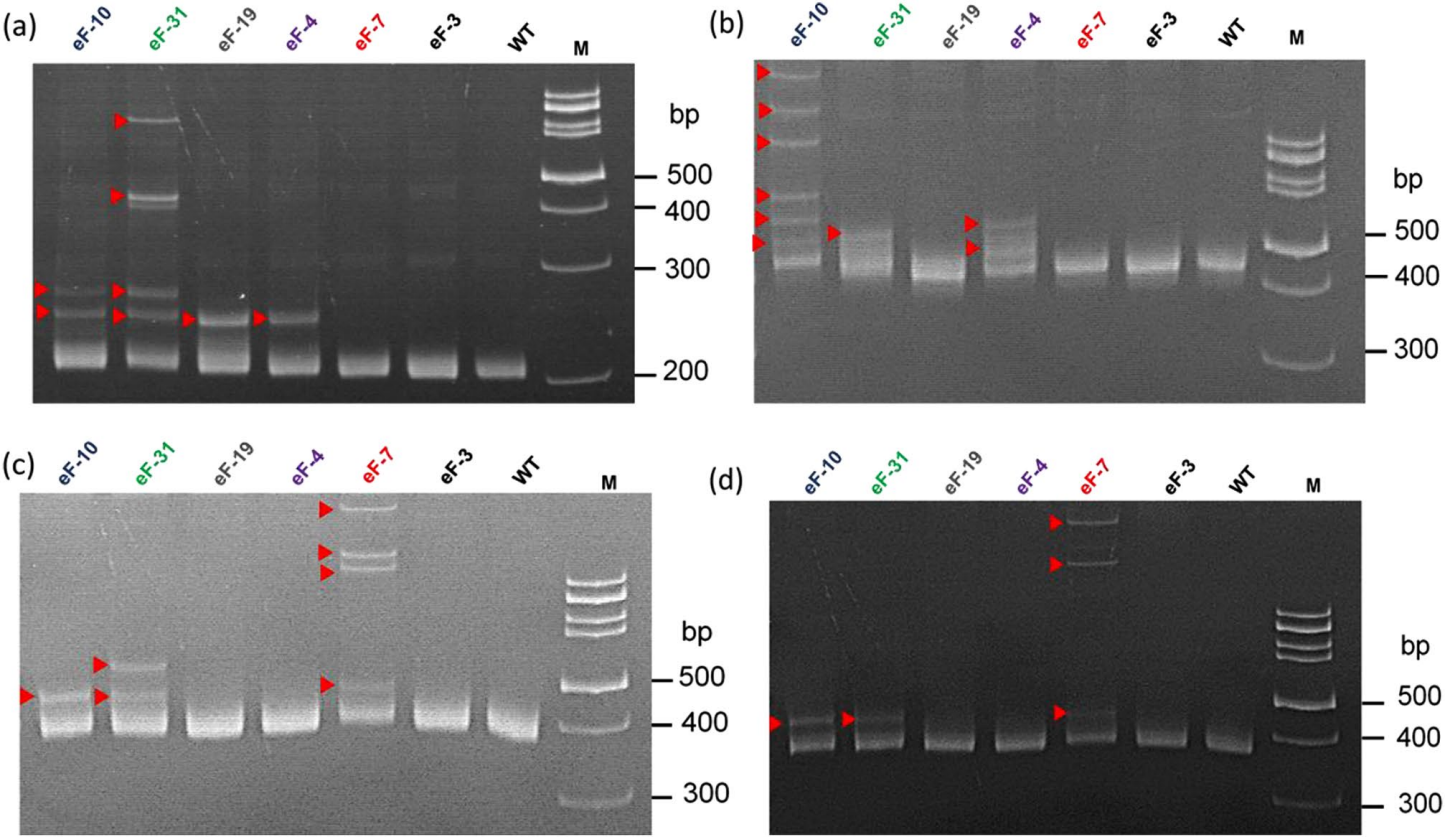
Identification of CRISPR/Cas9-induced mutations in the *eIF4E* genes by heteroduplex analysis. (**a**–**d**) PAGE-based heteroduplex analysis of *eIF4E1-S*, *eIF4E1-T*, *eIF4E2-S* and *eIF4E2-T*, respectively. eF-3–eF-31: T0 tobacco mutant lines. *WT* wild type plant, *M* marker 100 bp, Thermo Scientific. Red triangles denote heteroduplex bands.

To generate *eIF4E* mutants, we used tobacco leaf explants and the AGL1 strain harboring the pKSE401/4E1-sgRNA/4E2-sgRNA plasmid for *Agrobacterium-*mediated transformation. After shoot and subsequent root induction, we screened the regenerated plants for the presence of *cas9* and *nptII* genes by PCR (Table S2). Of the thirty transgenic T0 lines referred to as eFs, twenty-six yielded the expected PCR products for both *cas9* (711 bp) and *nptII* (766 bp), which were then subjected to heteroduplex analyses. We observed DNA band shifts for at least one of the Cas9-targeted *eIF4E* loci in each plant indicating that our CRISPR reagents were highly efficient in making independent DNA edits at multiple loci. We then selected five plants for detailed analysis (Fig. 2). Our data revealed that eF-19 carried mutations only in the *eIF4E1-S* gene. In contrast, eF-4 and eF-7 had mutations in both paralogs (T and S) of *eIF4E1* and *eIF4E2*, respectively. Importantly, eF-10 and eF-31 showed induced mutations at all four tested *eIF4E* genes by heteroduplex analysis (Fig. 2).

**Figure 3:**
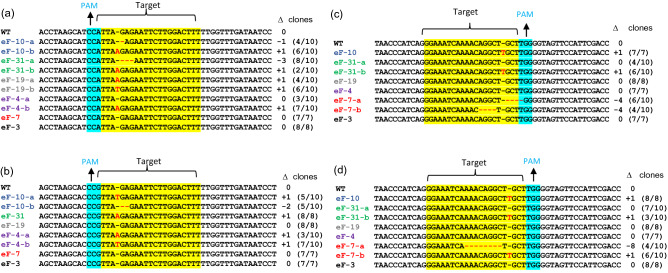
Alignment of *eIF4E* sequences isolated from the selected T0 lines. (**a**) *eIF4E1-S*, (**b**) *eIF4E1-T*, (**c**) *eIF4E2-S*, (**d**) *eIF4E2-T* loci. The sgRNA target sequences and the PAM motifs are highlighted in yellow and blue, respectively. Red letters indicate inserted nucleotides. Red dashed lines represent deleted nucleotides. Δ refers to changes in the CRISPR/Cas9 targeted sequence: 0, no change; − deletion; + insertion. “Clones” indicate the number of clones used for sequencing.

Next, we characterized the CRISPR/Cas9-induced mutations at each target loci by Sanger sequencing (Fig. 3). In agreement with the literature, we found both insertions and deletions at the CRISPR cut sites. All detected insertions were limited to 1 bp, however, deletions ranged from − 1 to − 8 bp. Interestingly, we observed biallelic and homozygous mutations at high frequency in the T0 lines (Fig. 3 and Table 1) suggesting that the mutations occurred very early, presumably at the single-cell stage of the regeneration process. Based on the deduced amino acid sequence analysis, most biallelic and homozygous mutations result in frameshifts with premature stop codons, and consequently produce truncated non-functional eIF4E proteins. Our sequencing data revealed that the eF-19 line harbors biallelic nonsense mutations in the *eIF4E1-S* gene only (Table 1). The eF-4 line carries biallelic null mutations in the *eIF4E1-T* gene and it is heterozygous for *eIF4E1-S*. eF-7 contains loss-of-function mutations both in the *eIF4E2-S* and *eIF4E2-T* genes in biallelic forms. In the eF-31 line, the Cas9-induced mutations disrupted both paralogs of the *eIF4E2* and *eIF4E1-T* genes, but *eIF4E1-S* gene may still be functional due to a 3 bp deletion resulting in a single amino acid loss in the eIF4E protein sequence. Importantly, eF-10 harbors null mutations in all four Cas9-targeted *eIF4E* genes (Table 1).

**Table 1:** Genotype of mutant lines used for PVY infection.

Codes	Genotypes	Targeted genes					
		eIF4E1-S	eIF4E1-T	eIF4E2-S	eIF4E2-T	eIF(iso)4E-S	eIF(iso)4E-T
isoE	iso4E-sstt	WT	WT	WT	WT	Homo	Homo
eF-10	E1-ss’tt’-E2-sstt	Biallelic	Biallelic	Homo	Homo	WT	WT
eF-31	E1-ss’tt/E2-SsTt	Biallelic^a^	Homo	Hetero	Hetero	WT	WT
eF-19	E1-ss’TT/E2-SSTT	Biallelic	WT	WT	WT	WT	WT
eF-4	E1-Sstt’/E2-SSTT	Hetero	Biallelic	WT	WT	WT	WT
eF-7	E1-SSTT/E2-ss’tt’	WT	WT	Biallelic	Biallelic	WT	WT
WT	E1-SSTT/E2-SSTT	WT	WT	WT	WT	WT	WT

^a^Sanger sequencing revealed a null mutation in one allele and a single-amino-acid deletion in the other, resulting in a partial loss of gene function.

### Assessing PVY resistance in the *eIF4E* edited T0 tobacco lines

**Figure 4:**
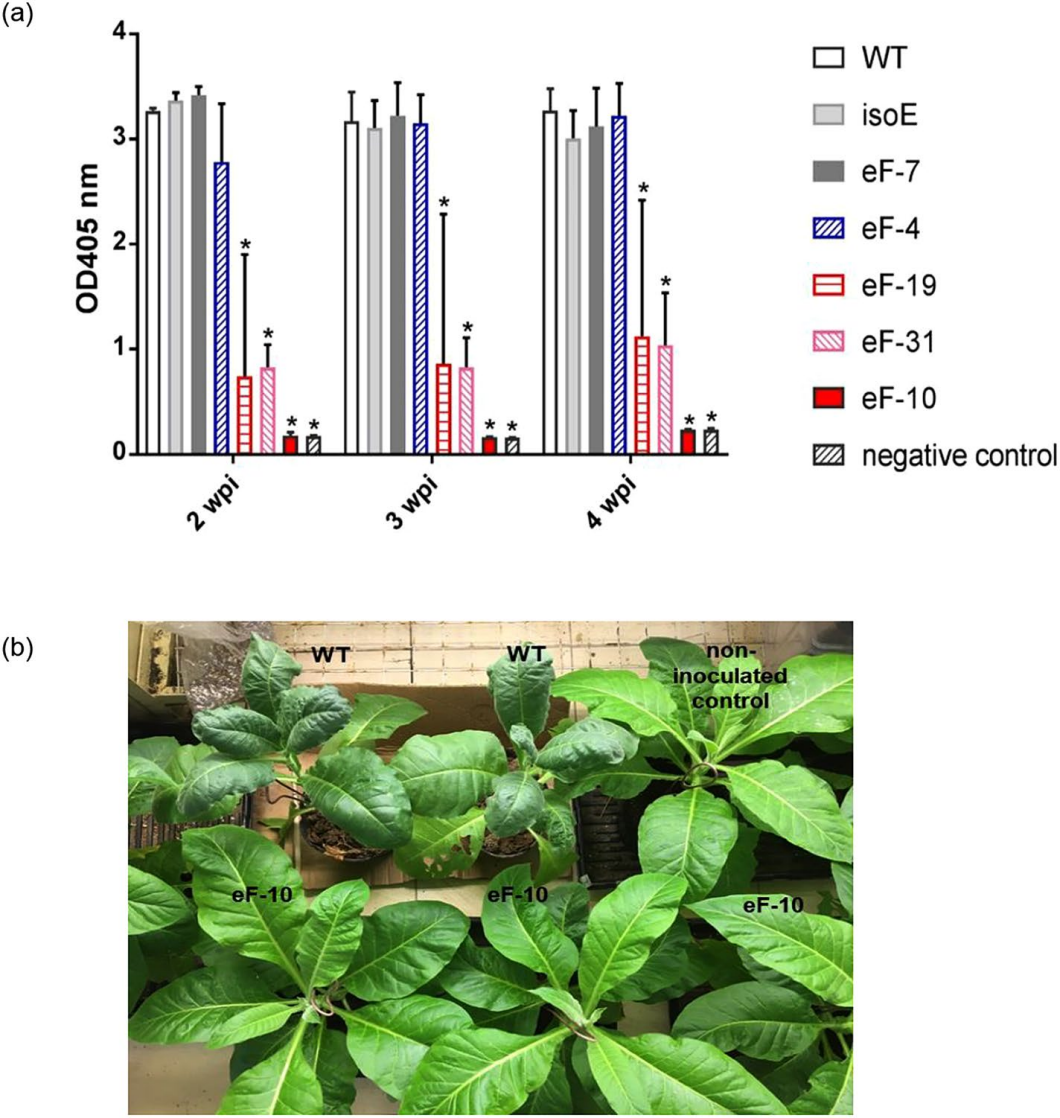
Effect of *eIF4E/eIF(iso)4E* mutations on PVY accumulation in T0 tobacco plants. (**a**) PVY^O^ accumulation in the systemic leaves of infected plants was assessed by DAS-ELISA at 2, 3 and 4 weeks post-inoculation (wpi); *WT* wild type plant, *isoE* *eIF(iso)4E-S* and *eIF(iso)4E-T* double mutant line, *eF-7* *eIF4E2-S* and *eIF4E2-T* double mutant line, *eF-4* *eIF4E1-T* mutant line, *eF-19* *eIF4E1-S* mutant line, *eF-31* *eIF4E1-S* and *eIF4E1-T* double mutant line; *eF-10* *eIF4E1-S*, *eIF4E1-T*, *eIF4E2-S* and *eIF4E2-T* quadruple mutant line; negative control: non-inoculated plant; Mean and standard errors of 405 nm absorbance (A405) values were calculated for 5 independent plants per line, *P < 0.05 versus corresponding positive control (WT). (**b**) PVY^O^-infected *Nicotiana tabacum* K326 plants at 10 wpi.

To investigate the impact of *eIF4E* gene redundancy on PVY susceptibly, we decided to challenge the T0 lines carrying a variety of homo- and heterozygous mutations in the *eIF4E* gene family including eF-19 (E1-ssTT/E2-SSTT), eF-4 (E1-Sstt/E2-SSTT), eF-31 (E1-sstt/E2-SsTt), eF-7 (E1-SSTT/E2-sstt) and eF-10 (E1-sstt/E2-sstt) with a local PVY^O^ isolate. We also added a tobacco line harboring null mutations in the *eIF(iso)4E* gene family (iso4E-sstt)^47^ as control (Supplementary Table S1, Fig. 4a). To assess PVY resistance in the selected tobacco lines, we employed DAS-ELISA, a widely used and highly accurate technique for immunodetection of virus infections^15,35,48,49^. Two weeks after rub inoculation, PVY accumulation was detected in the systemic leaves of eF-7, eF-4 and isoE (iso4E-sstt) lines at similar level to WT plants (Fig. 4a, Supplementary Table S1). In contrast, the eF-19 and eF-31 lines showed lower PVY^O^ titer, even 4 weeks post infection (Fig. 4a). However, only a small fraction of eF19 plants displayed systemic accumulation throughout the experiment (Supplementary Table S1) suggesting that PVY resistance is more durable in eF19 then in eF-31. More importantly, no PVY^O^ infection was detected in the eF-10 mutant lines at any time points tested (Fig. 4a,b and Supplementary Table S1).

### Investigating PVY resistance in the segregating T1 offspring of eF-10

**Figure 5:**
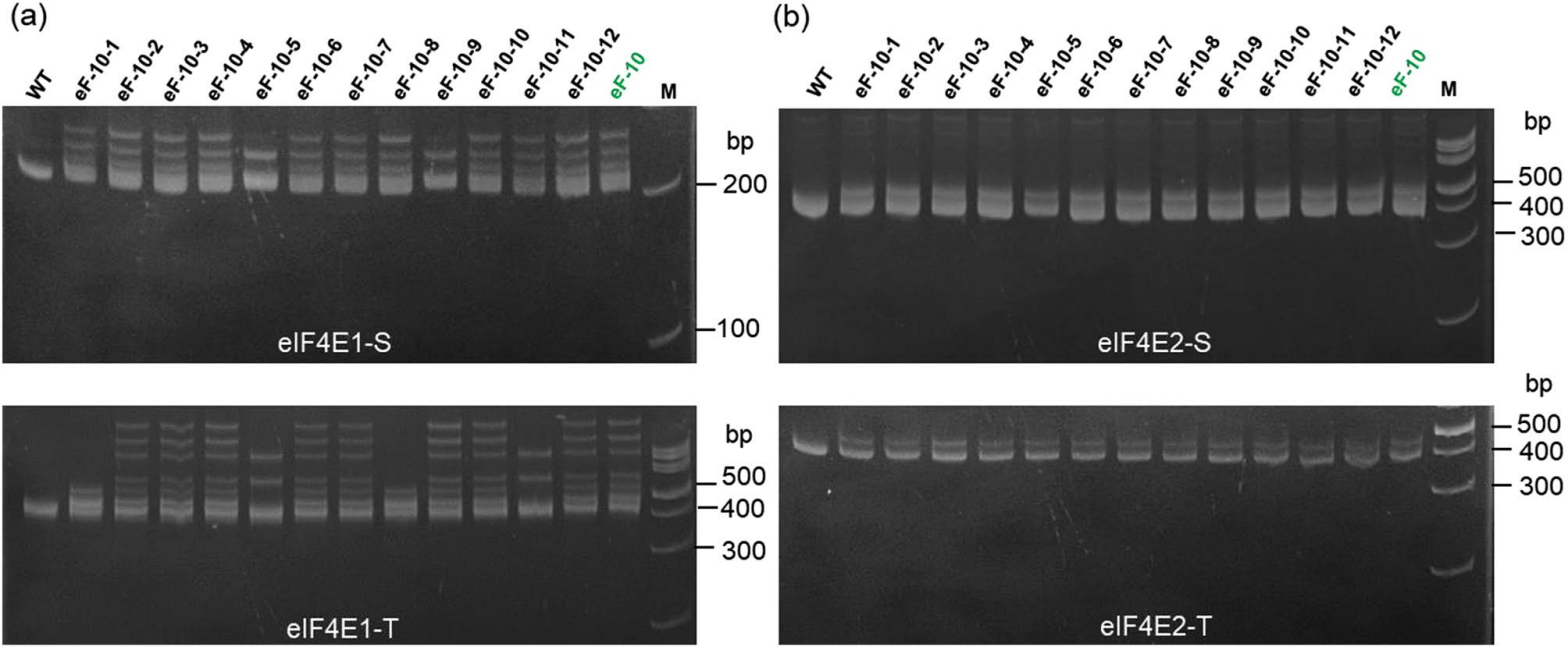
Inheritance of induced mutations at T1 generation of the eF-10 mutant line. (**a**, **b**) PAGE-based heteroduplex analysis of *eIF4E1* and *eIF4E2*, respectively; eF-10: T0 mutant line. eF-10–1–eF-10–12: T1 offspring of T0 eF-10 line. *WT* wild type plant, *M* marker 100 bp, Thermo Scientific.

To further confirm the genotype of eF-10 and examine the effect of *eIF4E* mutations on PVY^O^ resistance in tobacco, we analyzed the inheritance of Cas9-induced mutations and PVY resistance in the segregating T1 progeny. First, we genotyped twelve randomly selected seedlings by heteroduplex analysis (Fig. 5). No band shift was detected for both *eIF4E2-S* and *eIF4E2-T* in the offspring (Fig. 5b), which confirms that eF-10 harbored homozygous mutations for *eIF4E2s* in T0. As expected, we observed segregating DNA bands for the *eIF4E1-S* (eF-10–5 and eF-10–9, Fig. 5a) and *eIF4E1-T* (eF-10–5, eF-10–11, eF-10–1 and eF-10–8; Fig. 5a) genes indicating that both *eIF4E1s* indeed contained biallelic mutations in T0. Sequencing of the corresponding loci from selected eF-10 T1 plants (Supplementary Fig. S5) revealed that eF-10–5 and eF-10–9 carried one of the insertion (+ 1 bp) and deletion (-1 bp) alleles of the *eIF4E1-S* gene, respectively, while eF-10–1 and eF-10–8 inherited both mutant alleles of *eIF4E1-T* from the T0 generation (Supplementary Fig. S5a). Similarly, biallelic mutations (− 2 and + 1 bp) of *eIF4E1-T* were inherited from T0 plants in eIF-10–2 and eIF-10–6, whereas eF-10–5, eF-10–11, eF-10–1 and eF-10–8 were homozygous for the *eIF4E1-T* mutant alleles (Supplementary Fig. S5b). In addition, 1 bp insertion was detected in the *eIF4E2-S* and *eIF4E2-T* genes in all sequenced T1 plants (Supplementary Fig. S5c,d). All in all, our sequencing data were in line with the heteroduplex analysis and confirmed the presence of loss of function mutations in *eIF4E1-S, eIF4E1-T, eIF4E2-S* and *eIF4E2-T* in eF-10 and its progeny.

**Figure 6:**
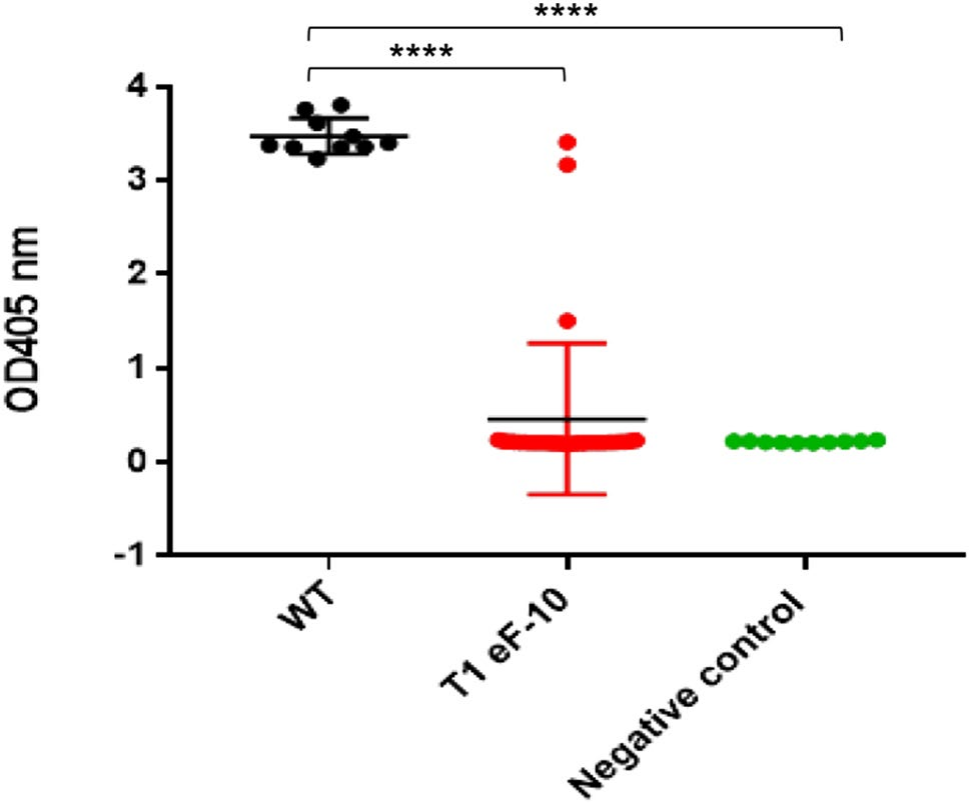
Analysis of PVY accumulation in T1 generation of the eF-10 line. PVY^O^ accumulation in the systemic leaves of infected plants was assessed by DAS-ELISA at 4 weeks post-inoculation; *WT* wild type plant, *T1 eF-10* T1 progeny of eF-10 line; negative control: non-inoculated plant. Mean and standard errors of 405 nm absorbance (A405) values were calculated for 30 (T1 eF-10) or 10 (WT and negative control) independent plants per line; ****P < 0.0001 versus corresponding positive control (WT).

To evaluate PVY resistance, we randomly selected thirty T1 offspring of eF-10 and rub inoculated them with PVY^O^. We then monitored the phenotype of virus infected plants and subsequently analyzed the virus load at 4 weeks post inoculation by DAS-ELISA in the newly emerging (systemic) leaves since this timepoint gave us the most reliable results in T0 (Fig. 4). As expected, all WT plants showed a typical PVY phenotype. However, none of the T1 progeny of eF-10 developed PVY symptoms. In agreement with the phenotypic observation, the vast majority of eF-10 T1s (27 out of 30, 90%) had similar DAS-ELISA values to the mock inoculated plants (Fig. 6). From these experiments we conclude that null mutations in all four *eIF4E* genes are associated with highly efficient and durable resistance to PVY^O^ in tobacco.

### Off-target analysis

Five potential off-target sites of 4E1-sgRNA and 4E2-sgRNA were predicted by CCTop in the *N. tabacum* genome (Supplementary Table S3). However, no Cas9-induced mutations were identified in T0 and its progeny by PAGE-based heteroduplex analysis and sequencing of the corresponding loci (Supplementary Fig. S6).

## Discussion

Both single and multiple sgRNA expressing CRISPR/Cas9 vectors can be utilized to edit several genes simultaneously that share high sequence complementarity with the first 20 nucleotide (nt) of sgRNA(s). In a recent report, a single sgRNA construct was designed to target a highly conserved region in the berberine bridge enzyme-like (*BBL*) gene family which is involved in nicotine biosynthesis, and nicotine‐free tobacco plants carrying loss-of-function mutations in all six *BBL* genes were obtained in the T3 generation^50^. Similarly, members of the *rbcS* gene family displaying high sequence homology were knocked out by a dual sgRNA-CRISPR/Cas9 vector in *Nicotiana tabacum* cv. Petit Havana^51^. Moreover, multiple sgRNA expressing constructs were also employed to simultaneously edit non-homologous genes (phytoene desaturase (*PDS*) and PDR-type transporter (*PDR6*)) in tobacco^52^. In this study, we developed a dual sgRNA CRISPR/Cas9 system to induce mutations in two separate clades of the *eIF4E* gene family in the tobacco variety K326 (Figure S1), namely *eIF4E1* *(eIF4E1-S* and *eIF4E1-T*) and *eIF4E2* (*eIF4E2-S* and *eIF4E2-T*), respectively. We identified regenerated plants with loss-of-function mutations in the combinations of one, two, three and all four targeted genes in the T0 generation, indicating that CRISPR/Cas9 can be efficiently harnessed for precision combinatorial gene editing applications in tobacco.

eIF-mediated potyvirus resistance has been extensively studied in many plants^12,48,53,54^, which is based on the interaction of the viral genome-linked protein (VPg) with the eukaryotic translation initiation factor eIF4E or its orthologue eIF(iso)4E to initiate viral RNA translation and subsequent virus replication^55,56^. Consequently, down regulation of the above translation initiation factors or mutations that disrupt the binding of VPg to the corresponding eIFs could result in resistance to potyviruses^57,58^. Interestingly, we found that simultaneous loss-of function mutations in eIF4E2s and eIF(iso)4Es (eF-7 and isoE, respectively; Fig. 4a and Supplementary Table S1) and partial knock out of eIF4E1s (E1-Sstt’ in eF-4, Fig. 4a and Supplementary Table S1) had no impact on PVY^O^ resistance. In contrast, systemic accumulation of PVY^O^ was significantly reduced in the eF-31 line harboring partially disrupted *eIF4E1-S* and knocked out *eIF4E1-T* (Fig. 4a and Supplementary Table S1). In the eF-19 line, which carries null mutation only in *eIF4E1-S*, PVY^O^ was detected in the fraction of infected plants (20% and 40% at 2 and 4 wpi, respectively; Supplementary Table S1). Thus, our data indicate that *eIF4E1-S* is required but not sufficient for durable PVY^O^ resistance in K326. This is in line with previous reports revealing that tobacco plants carrying natural frameshift or nonsense mutations in *eIF4E1-S* were only partially resistant to a large collection of PVY isolates from the O and C clades^43^, and tobacco cultivars VAM and TN90, lacking *eIF4E1-S*, showed limited resistance to PVY^NTN^^38^.

Recent studies suggested that eIF4Es could act redundantly in PVY translation^17,38,43^. Conversely, more than one eIF4E orthologues may need to be deleted/mutated to generate efficient and stable PVY resistance. Indeed, in our study, the eF-10 line harboring quadruple nonsense mutations in all four *eIF4E* genes (*eIF4E1-S*, *eIF4E1-T*, *eIF4E2-S* and *eIF4E2-T*) exhibited complete and durable resistance to PVY^O^ both in the T0 and in the T1 generation (Figs. 4 and 6). Thus, our findings provide evidence for the above hypothesis. Since random mutations generated during virus replication may yield to novel VPg variants that can selectively recruit eIF4E orthologues or isoforms, simultaneous deletion of redundant viral host factors could prevent or at least reduce the efficacy of virus replication, and consequently suppress/delay the emergence of resistance breaking virus strains. Hence, our work may also establish a framework to create broad spectrum virus resistance in tobacco and in other crop species. Interestingly, a recent study by Udagawa et al.^38^ showed that simultaneous knock out of *eIF4E1-S and eIF(iso)4E1-T* resulted in high-level resistance against both PVY and RB-PVY in tobacco, which indicates that eIF(iso)4E1-T can also be recruited by PVY variants. Thus, further studies may be performed to evaluate PVY resistance in tobacco lines carrying mutations in all 5 genes *eIF4E1-S*, *eIF4E1-T, eIF4E2-S, eIF4E2-T and eIF(iso)4E1-T.*

## Materials and methods

### Identification and phylogenetic analysis of the *eIF4E* gene family in *N. tabacum* K326

To identify the members of the *eIF4E* gene family in *N. tabacum* K326^41^, the IF4E domain (pfam01652) of the tobacco eIF4E amino acid sequence QNT12790.1 (GenBank) was used as a protein query in TblastN search using the Sol Genomics website (https://solgenomics.net/). The returned sequences were then subjected to Pfam analysis (http://pfam.xfam.org/) using the default parameters to validate the *eIF4E* genes. Next, a phylogenetic analysis was conducted to investigate the evolutionary relationship among the *eIF4E* gene family members in *N. tabacum* K326 and in its *Nicotiana* ancestors, *N. sylvestris* and *N. tomentosiformis*. To this end, the coding DNA Sequences (CDS) were aligned by ClustalW (MEGA 6.0 software)^59^ and the phylogenetic tree was then generated by the maximum-likelihood method based on the JTT model using 1000 replicate bootstrap support.

### Single guide RNA (sgRNA) design and CRISPR/Cas9 binary vector construction

To generate Cas9‐induced mutations in all four *eIF4E* homologs, two independent Cas9 target sites referred to as 4E1-gRNA and 4E2-gRNA were identified by CCTop (https://cctop.cos.uni-heidelberg.de:8043/)^60^. Next, complementary single-stranded DNA oligos corresponding to 4E1-gRNA and 4E2-gRNA (Supplementary Table S2) were annealed and subsequently ligated into the *Bsa* I-digested binary vector pKSE401 (Addgene, #62202)^61^ to generate two single guide RNA constructs pKSE401/4E1-sgRNA and pKSE401/4E2-sgRNA, respectively. Finally, the sgRNA expression cassettes were recombined into a single construct pKSE401/4E1-sgRNA/4E2-sgRNA by Golden Gate cloning^62^. The sequence of the pKSE401/4E1-sgRNA/4E2-sgRNA plasmid was confirmed by Sanger sequencing, which was subsequently introduced into *A. tumefaciens* strain AGL1 for tobacco transformation.

### *Agrobacterium*-mediated tobacco transformation and transgene confirmation

*N. tabacum* cultivar K326 (provided by Tobacco Institute One Member Comapy Limited, Vietnam) was used for *Agrobacterium*-mediated transformation following the protocol by Topping et al.^63^. Briefly, a single *Agrobacterium* colony harboring the pKSE401/4E1-sgRNA/4E2-sgRNA binary plasmid was cultured in 50 mL of LB medium containing appropriate antibiotics at 200 rpm at 28 °C for 14–16 h until OD_600_ reached 0.6–0.8. The bacterial cells were then harvested by centrifugation at 5000 rpm for 10 min at room temperature and then resuspended in ½ MS liquid medium supplemented with 0.02 µM acetosyringone. The leaves of in vitro grown tobacco plants were cut into small pieces (1 × 1 cm) and immersed in the *Agrobacterium* suspension for 30 min with gentle agitation. The infected explants were then placed onto the co-cultivation medium and kept in the dark for 2 days at 25 ± 2 °C. Next, the explants were washed with sterile water supplemented with 500 mg/L cefotaxime. After removing the excess water by sterile filter papers, the leaf pieces were cultured on selection medium containing 150 mg/L kanamycin and 500 mg/L cefotaxime. Healthy shoots regenerated from transformed explants were transferred to rooting medium. Rooted tobacco plants with 3–4 leaves were used to identify the presence of the CRISPR/Cas9 construct by PCR using primer pairs specific for *cas9* and *nptII* genes (Supplementary Table S2).

### Identification and characterization of CRISPR/Cas9-induced mutations

Genomic DNA was extracted from leaves of wild-type (WT) and transgenic tobacco plants using a CTAB-based extraction method^64^. DNA spanning the Cas9 gRNA target sites was amplified by PCR using *eIF4E*-specific primer pairs listed in Table S2. CRISPR/Cas9-induced mutations were detected by heteroduplex analysis using native polyacrylamide gel electrophoresis (PAGE) as previously described^65^. Briefly, the mixture containing equal amounts of PCR products amplified from WT and the corresponding transgenic tobacco line was denatured at 95 °C for 10 min, which was then subsequently cooled down to room temperature for renaturation. Next, DNA was separated in 15% native polyacrylamide gels. Note, CRISPR/Cas9-induced indels result in shifted DNA bands due to heteroduplex formation with WT DNA^65^. To further characterize the Cas9-induced mutations, the PCR amplicons were purified by the ProNex® Size-Selective Purification System (Promega, USA) and then ligated into the pJET1.2/blunt cloning vector (Thermo Fisher Scientific, USA). Seven to ten clones from each line were sequenced by the Sanger method using the Applied Biosystems 3500 Genetic Analyzer (Thermo Fisher Scientific, USA). The sequencing data were analyzed by BioEdit (v. 7.2.5, Ibis Biosciences, Carlsbad, CA, USA)^66^. The stability of the Cas9-induced mutations was assessed at three time points in every transgenic tobacco line, first in in vitro culture and then 2 and 4 weeks after transferring the rooted plants into soil.

The selected T0 plants harboring mutations in the CRISPR/Cas9-targeted *eIF4E* genes were grown in the greenhouse. After self-pollination, the seeds were collected and subsequently germinated on a substrate containing perlite and vermiculite (1:3 v/v) and the seedlings were then transferred to the greenhouse. The segregating T1 plants were characterized for the presence of Cas9-induced mutations in the *eIF4E* genes as described above.

### Potential off-target analysis

Potential off-target sites were predicted by CCTop. To assess the frequency of off-target mutations, the genomic DNA isolated from the T0 mutant lines and their T1 progeny were pooled together, and then used to amplify and sequence the putative off-target sites using locus-specific primers (Supplementary Table S2).

### PVY isolation and multiplication

To obtain PVY isolates for the virus challenge experiments, potato plants showing PVY symptoms were collected from different locations and the presence of PVY was confirmed by RT-PCR using PVY capsid protein (CP)-specific primers (Supplementary Table S2). Sequencing and phylogenetic analysis of the collected PVY isolates (Supplementary Fig. S3) revealed that the majority of potato plants were infected with PVY^O^—the most common PVY strain with a wide host range^67^. The selected PVY isolate was multiplied and maintained in virus-free *Nicotiana benthamiana* plants (a gift from Dr. H.T. Phan, Leibniz Institute of Plant Genetics and Crop Plant Research, Gatersleben, Germany) as described by Korbecka-Glinka^68^ with minor modifications. Briefly, PVY-infected potato leaves were homogenized in ten volumes of 0.01 M phosphate-buffered saline (PBS) pH 7.4 using a cold mortar and pestle. The homogenate was then gently rubbed onto *N. benthamiana* leaves, which were dusted with silicon carbide powder prior infection. Next, the excess carborundum and sap were removed by rinsing the leaves with distilled water. Inoculated plants were kept in the greenhouse at 22 °C (± 2 °C) under long day conditions, which were sheltered from direct sunlight for 48 h. Two weeks post infection, PVY symptoms were observed on inoculated *N. benthamiana* plants (Supplementary Fig. S4). The presence of PVY^O^ was confirmed by RT-PCR as described above.

### PVY susceptibility test of the *eIF4E* mutant tobacco lines

Selected T0 lines harboring mutations in *eIF4E* genes were propagated and multiplied in vitro and then transferred to the greenhouse for virus infection. Sap collected from PVY^O^-infected *N. benthamiana* leaves was rubbed onto the top leaves of at least five plants from each T0 line (genotype) in 5–6-leaf developmental stage as described above. Mock inoculated plants were used as negative controls. In T1 generation, up to 30 in vitro propagated plants were challenged with PVY^O^ from each segregating genotype. The presence and stability of the Cas9-induced mutations in *eIF4E* genes were investigated prior virus infection as described above. PVY^O^ accumulation was analyzed at two-, three- and four-weeks post-inoculation (wpi). Systemic leaves were sampled and subjected to double-antibody sandwich enzyme-linked immunosorbent assay (DAS-ELISA) using a commercially available kit (BIOREBA, Germany) according to the manufacturer’s instruction. The absorbance at OD_405_ was measured by a Benchmark microplate reader (Bio‐Rad). Data were plotted by the GraphPad Prism software (version 7.04; LA Jolla, California, USA). For statistical analysis, a one-way analysis of variance (ANOVA) was performed (P values < 0.05). Plant growth and PVY symptoms were continuously monitored up to 10 weeks post inoculation.

### Ethics declarations

We had obtained permission to collect plants. All the experiments were performed in accordance with relevant guidelines and regulations.

## Supplementary Information

Supplementary Information.

## Data Availability

The data that support the findings of this study are openly available in GenBank of NCBI at https://www.ncbi.nlm.nih.gov/, Reference Number KM202067, KM202068, KM202069, KM202070, KM202065, KF155696, KM202071, and KM202066.
