# Supplementary material for: Simultaneously induced mutations in eIF4E genes by CRISPR/Cas9 enhance PVY resistance in tobacco
Source: Sci Rep. 2022 Aug 26;12:14627. doi: 10.1038/s41598-022-18923-0 (PMC9418239; doi:10.1038/s41598-022-18923-0)
Supplement: Supplementary file 1 — Supplementary Information. [file 41598_2022_18923_MOESM1_ESM.pdf]

Simultaneously induced mutations in *eIF4E* genes by CRISPR/Cas9 enhance PVY resistance in tobacco

Ngoc Thu Le[1,2], Huyen Thi Tran[1], Thao Phuong Bui[1], Giang Thu Nguyen[1], Doai Van Nguyen[1], Dong Thi Ta[1], Duy Dinh Trinh[1], Attila Molnar[3], Ngoc Bich Pham[1,2], Ha Hoang Chu[1,2*], Phat Tien Do[1,2*]

Supplementary Information

Supplementary figures:

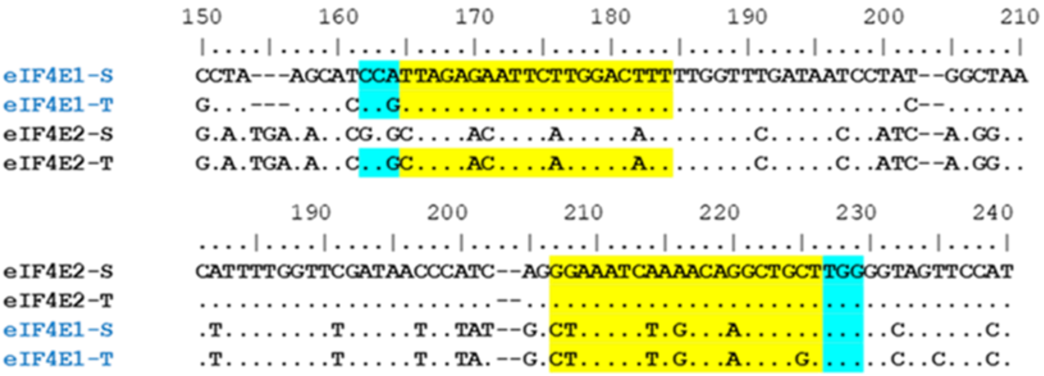

Supplementary Figure S1. Target specificity of selected sgRNAs. Sequence alignment of genes shown in Figure 1b. Top panel: 4E1-gRNA targeted region, bottom panel: 4E2-gRNA targeted region. The CRISPR/Cas9-targeted sequences and the PAM motifs are highlighted in yellow are blue, respectively. Dots represent identical nucleotides.

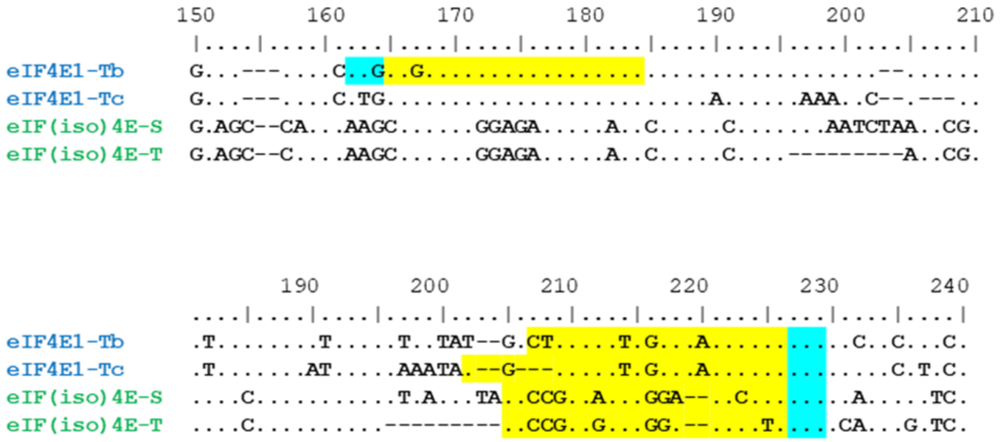

Supplementary Figure S2. Nucleotide sequence alignment of the non-targeted *eIF4E* genes. Top panel: 4E1-gRNA targeted region, bottom panel: 4E2-gRNA targeted region. The sgRNA targeted sequences and the PAM motifs are highlighted in yellow and in blue, respectively. Dots indicate identical nucleotides.

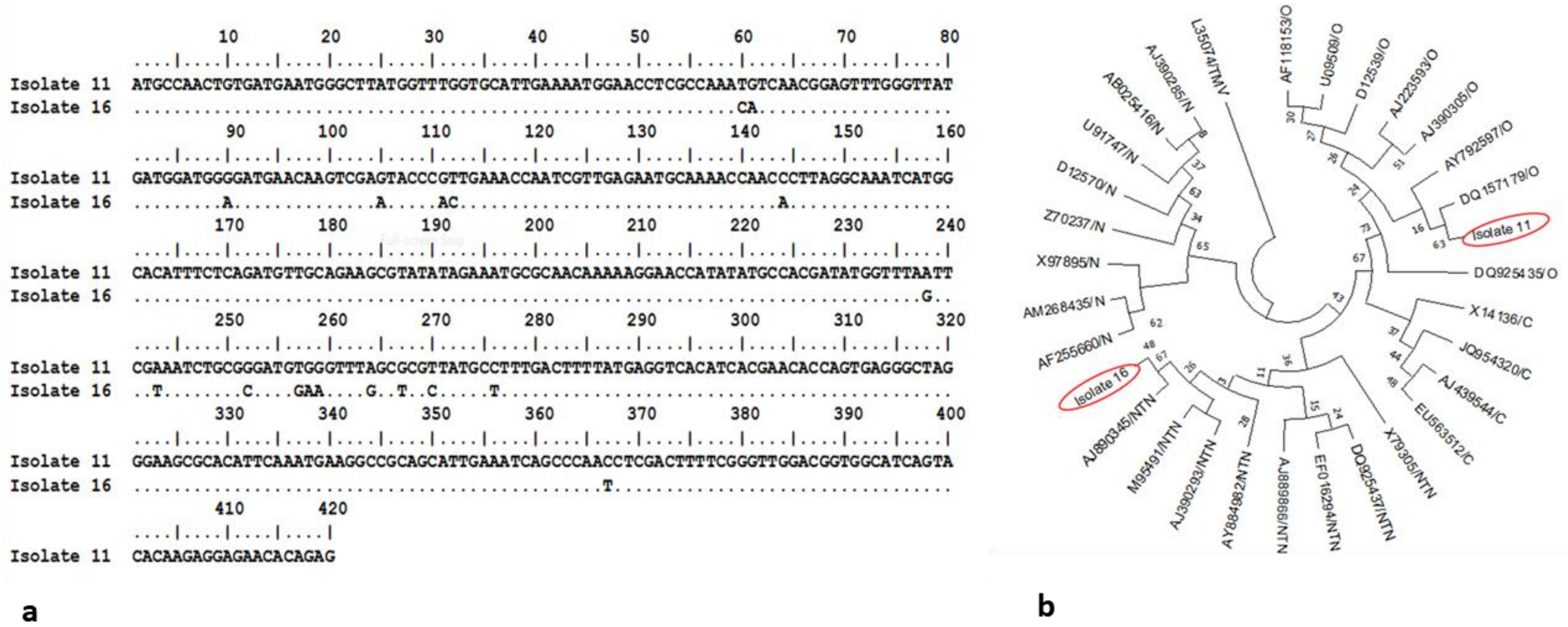

Supplementary Figure S3. Analysis of coat protein (CP) sequences of different PVY isolates collected from infected potato plants. (a) Nucleotide alignment of the CP sequence of PVY isolates 11 and 16. (b): Phylogenetic tree of the CP sequences of PVY isolates, which is constructed by MEGA6 using the maximum-likelihood method (1,000 replicates).

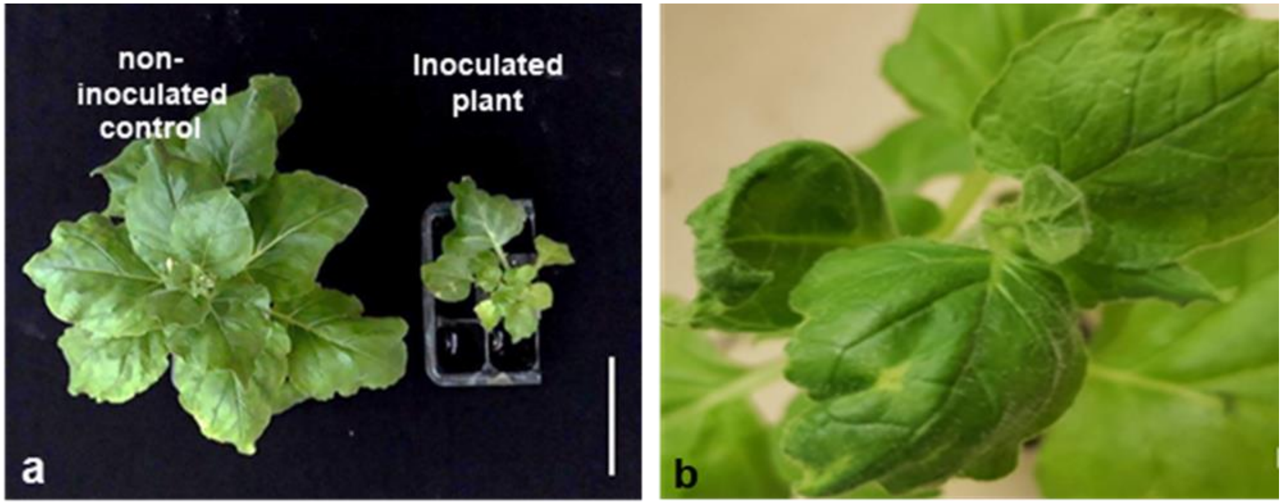

Supplementary Figure S4. (a, b) PVY^{O} symptoms on *Nicotiana benthamiana* at 3 weeks post inoculation (wpi).

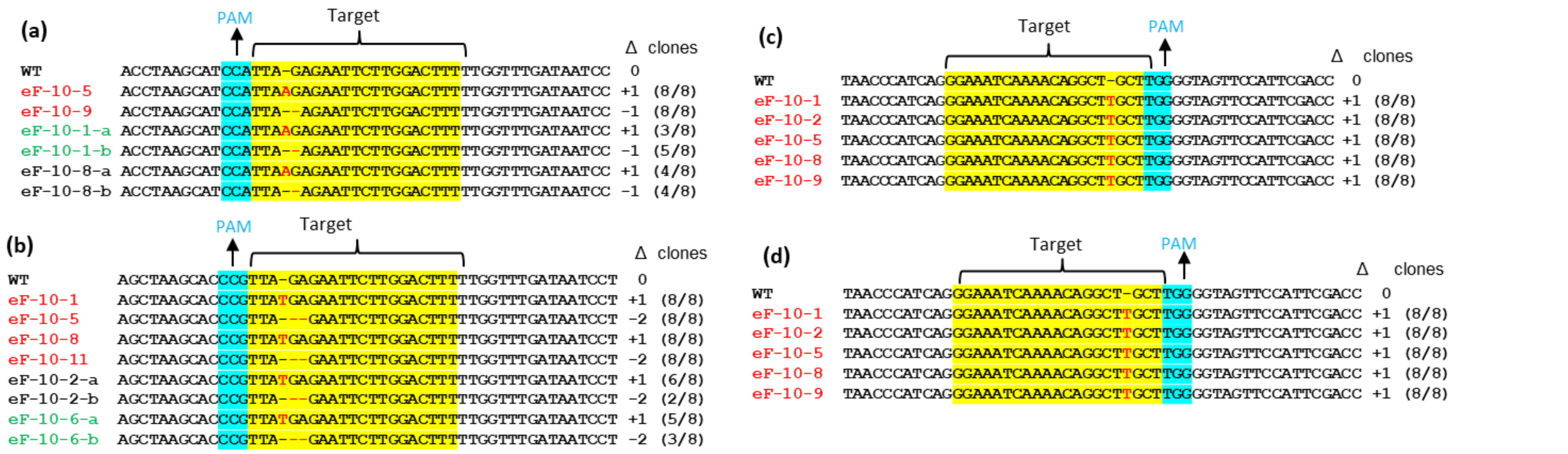

Supplementary Figure S5. Inheritance of CRISPR/Cas9-induced mutations in the T1 progeny of the eF-10 line. (a) *eIF4E1-S*, (b) *eIF4E1-T*, (c) *eIF4E2-S*, (d) *eIF4E2-T* loci. Labeling as of Figure 3.

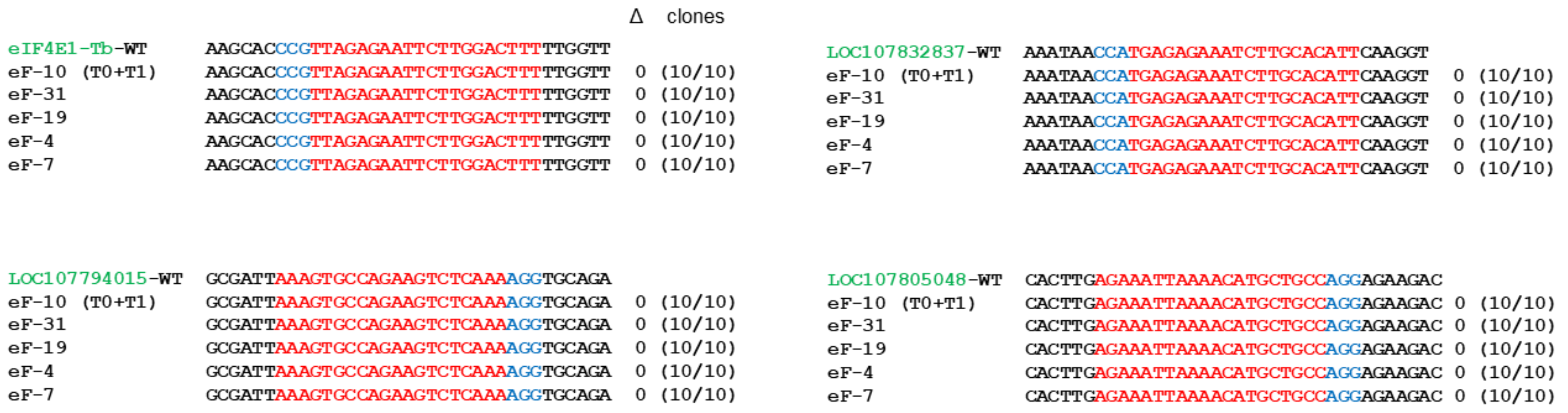

Supplementary Figure S6. Sequence analysis of potential off-target sites. The sgRNA targeted sequences and the PAM motifs are highlighted in red and blue, respectively. Δ refers to changes in the CRISPR/Cas9-targeted sequences: 0, no change.

Supplementary tables:

Supplementary Table S1. Summary of PVY infection in different eIF4E mutant genotypes

Tobacco line	Genotype of eIF4E/eIF(iso)4E	PVY infected plants confirmed by DAS-ELISA		
		**2 wpi**	**3 wpi**	**4wpi**
isoE	iso4E-sstt	5/5 (100%)	5/5 (100%)	5/5 (100%)
eF-10	E1-ss’tt’-E2-sstt	0/5 (0%)	0/5 (0%)	0/5 (0%)
eF-31	E1-ss’tt/E2-SsTt	5/5 (100%)	5/5 (100%)	5/5 (100%)
eF-19	E1-ss’TT/E2-SSTT	1/5 (20%)	2/5 (40%)	2/5 (40%)
eF-4	E1-Sstt’/E2-SSTT	5/5 (100%)	5/5 (100%)	5/5 (100%)
eF-7	E1-SSTT/E2-ss’tt’	5/5 (100%)	5/5 (100%)	5/5 (100%)
WT	E1-SSTT/E2-SSTT	5/5 (100%)	5/5 (100%)	5/5 (100%)

wpi: weeks post-inoculation

Supplementary Table S2. Primers used in this study

Oligonucleotide	Sequence (5’-3’)	Description
4E1-gRNA_F	**ATTG**AAAGTCCAAGAATTCTCTAA	Annealed to creat 4E1-sgRNA
4E1-gRNA_R	**AAAC**TTAGAGAATTCTTGGACTTT	
4E2-gRNA_F	**ATTG**GGAAATCAAAACAGGCTGCT	Annealed to creat 4E2-sgRNA
4E2-gRNA_R	**AAAC**AGCAGCCTGTTTTGATTTCC	
cas9_F	ATCGGCATTCCATCAAGAAG	Amplifying a fragment of *cas9* gene
cas9_F	CCAGGTCATCGTCGTATGTG	
nptII_F	AGATGGATTGCACGCAGGTT	Amplifying a fragment of *nptII* gene
nptII_R	AAGGCGATAGAAGGCGATGC	
BsaI/4E1-gRNA_F	ATATATGGTCTCGATT*G*AAAGTCCAAGAATTCTCTAA	Amplifying the cassette 4E1-sgRNA_AtU6 terminator
U6ter/BsaI_R	ATATATGGTCTCGATG*G*TATTGGTTTATCTCATC	
BsaI/U6p_F	ATATATGGTCTC*G*CCATTCGACTTGCCTTCCGC	Amplifying the cassette AtU6 promoter_4E2-sgRNA
4E2-gRNA/BsaI_R	ATTATTGGTCTC*G*AAACAGCAGCCTGTTTTGATTTCC	
4E1-S-exon1_F	CCTGAAGAAGGAGAAATTGTGGAT	Amplifying 1th exon of eIF4E1-S (201 bp)
4E1-exon1_R	AACTTACCCCCAAAAATCTTCGA	
4E1-T-exon1_F	CTATTACTAAAAAGCAAGCTACAACC	Amplifying 1th exon of eIF4E1-T (425 bp)
4E1-exon1_R	AACTTACCCCCAAAAATCTTCGA	
4E2-exon1_F	CATTTTCAATTGAAACCCATCACCA	Amplifying 1th exon of eIF4E2-S (364 bp)
4E2-S-exon1_R	CAAAGTAAGGGGTAGCGTACGG	
4E2-exon1_F	CATTTTCAATTGAAACCCATCACCA	Amplifying 1th exon of eIF4E2-T (372 bp)
4E2-T-Exon1_R	TTTCTAGCAAGAACTCGGTCGAAT	
4E1-Tb-exon1_F	CATAACACCAAAATGGCAGG	Amplifying 1th exon of eIF4E1-Tb (310 bp)

4E1-Tb-exon1_R	CGAGAAATATGAAATACTCAAATACG	
LOC107794015_F	TTGAGAAATGCGCGATTGAC	Amplifying flanking region of potential off-target site on LOC107794015 (311 bp)
LOC107794015_R	CCTGCAAGTCTCAGCCACTC	
LOC107832837_F	CCCTGTCGTCTCACACCAAC	Amplifying flanking region of potential off-target site on LOC107832837 (328 bp)
LOC107832837_R	CAGCAGCAGCCTCATTGAAA	
LOC107805048_F	TACATGTCTAAATTTGTAACAAGCATC	Amplifying flanking region of potential off-target site on LOC107805048 (300 bp)
LOC107805048_R	TTCATTTGTAGTTTGGTCTTCAGC	

The sequences in bold represent 5'-flanking nucleotides, and the BsaI restriction enzyme recognition site is underlined.

Supplementary Table S3. Summary of off-target analysis

	**Sequence**	**No. of mismatching bases**	**Gene locus**	**Region**	**Presence of mutant**
**Target sequence**	AAAGTCCAAGAATTCTCTAACGG				
Potential off-target	AAAGTCCAAGAATTCTCCAACGG	1	*eIF4E1-Tb*	exon	0
Potential off-target	AAAGTGCCAGAAGTCTCAAAAGG	4	LOC107794015	exon	0
Potential off-target	AATGTGCAAGATTTCTCTCATGG	4	LOC107832837	exon	0
**Target sequence**	GGAAATCAAAACAGGCTGCTTGG				
Potential off-target	AGAAATTAAAACATGCTGCCAGG	4	LOC107805048	exon	0

Red letters indicate mismatched nucleotides; underlined letters indicate PAM
